# *Mycobacterium tuberculosis* infection up-regulates MFN2 expression to promote NLRP3 inflammasome formation

**DOI:** 10.1074/jbc.RA120.014077

**Published:** 2020-10-16

**Authors:** Fang Xu, Hui Qi, Jieqiong Li, Lin Sun, Juanjuan Gong, Yuanying Chen, Adong Shen, Wei Li

**Affiliations:** 1 Beijing Key Laboratory for Respiratory and Infectious Diseases, Beijing Pediatric Research Institute, Beijing Children's Hospital, Capital Medical University, National Center for Children's Health, Beijing, China; 2 Beijing Key Laboratory for Genetics of Birth Defects, Beijing Pediatric Research Institute, Beijing Children's Hospital, Capital Medical University, National Center for Children's Health, Beijing, China

**Keywords:** cytokine, microarray, mitochondria, mitofusin 2, Mycobacterium tuberculosis, NLRP3 inflammasome, cytokine, inflammasome, microarray, mitochondria

## Abstract

Tuberculosis (TB), caused by the infection of *Mycobacterium tuberculosis* (MTB), is one of the leading causes of death worldwide, especially in children. However, the mechanisms by which MTB infects its cellular host, activates an immune response, and triggers inflammation remain unknown. Mitochondria play important roles in the initiation and activation of the nucleotide-binding oligomerization domain-like receptor with a pyrin domain 3 (NLRP3) inflammasome, where mitochondria-associated endoplasmic reticulum membranes (MAMs) may serve as the platform for inflammasome assembly and activation. Additionally, mitofusin 2 (MFN2) is implicated in the formation of MAMs, but, the roles of mitochondria and MFN2 in MTB infection have not been elucidated. Using mircroarry profiling of TB patients and *in vitro* MTB stimulation of macrophages, we observed an up-regulation of MFN2 in the peripheral blood mononuclear cells of active TB patients. Furthermore, we found that MTB stimulation by MTB-specific antigen ESAT-6 or lysate of MTB promoted MFN2 interaction with NLRP3 inflammasomes, resulting in the assembly and activation of the inflammasome and, subsequently, IL-1β secretion. These findings suggest that MFN2 and mitochondria play important role in the pathogen-host interaction during MTB infection.

Tuberculosis (TB) is a common infectious disease caused by the infection of *Mycobacterium tuberculosis* (MTB). In a summary report from the World Health Organization in 2019, TB is one of the leading causes of death worldwide (1). As the pathogenesis of TB is very complex, it raises great difficulties in the precisely effective intervention of TB. Currently, pathogen-host interaction is an important hallmark for TB pathogenesis and progression. MTB invades host macrophages through various intercellular organelles to participate in various biological processes such as cellular energy metabolism, inflammatory response, and endocytosis.

Inflammasomes consist of NOD-like receptors (NLRs) containing caspase-recruitment domains (CARD), NALPs (NACHT-LRRs) containing pyrin domains (PYD), and NAIPs (neuronal apoptosis-inducing protein) containing BIR domains (2). Different NLRs associate with each other to form different inflammasome complexes in response to different pathogenic stimulating molecules or endogenous harmful signals (3). The nucleotide-binding oligomerization domain-like receptor with a pyrin domain 3 (NLRP3) inflammasome is a molecular platform activated upon signals of cellular “danger” to trigger innate immune defenses through the maturation of pro-inflammatory cytokines such as IL-1β (4). NLRP3 inflammasome is activated upon exposure to a broad range of signals, such as ATP (5), nigericin (6), fungi (7), bacteria that produce pore forming toxins (8), and viruses (9). There are several human diseases associated with inflammasome including TB. MTB infection associated with NLRP3 inflammasome requires the involvement of the bacterial virulence factor ESAT-6 (6-kDa early secretory antigenic target) (10). IL-1β plays a major role in host resistance to MTB (11). IL-1 receptor 1 is essential for IL-1–mediated signaling events in mycobacterial infection (12). Although it has been suggestive of a beneficial role of IL-1β in TB infectious diseases (13), compelling evidence has shown that excessive production of IL-1β is associated with more severe TB conditions and increased lung damage. Thus, how NLRP3 inflammasome is activated during MTB infection is important for understanding the pathogenesis and progression of TB.

Currently, studies have unveiled the pivotal roles of mitochondria in the initiation and activation of the NLRP3 inflammasome. Loss of mitochondrial membrane potential couples with NLRP3 inflammasome activation (14). It has been reported that NLRP3 inflammasome activation requires two signals (15). One is called initiator or primer that is activated through transcriptional regulation to induce NF-κB–dependent expression of both proIL-1β and NLRP3. Another is called activator by which NLRP3 inflammasome is assembled and activated to release IL-1β. However, the underlying mechanisms of NLRP3 inflammasome activation are not well understood. The reactive oxygen species (ROS) have been shown to play a role in the priming step. Mitophagy blockade leads to the accumulation of damaged mitochondria, and increases ROS production from mitochondria (14). In addition, mitochondrial membrane is involved in the initiation and regulation of an innate immune system (16). Mitochondria-associated endoplasmic reticulum membranes (MAMs) are the membranes that mitochondria physically interact with ER. MAMs play key roles in material transfer and signal transduction including Ca^2+^ signaling. Inactivated NLRP3 proteins reside mostly on the ER. Stimulated by activators, NLRP3 and ASC colocalize with MAMs in the perinuclear region (14, 17). Diacylglycerol rapidly accumulates in the Golgi apparatus and recruits protein kinase D. Protein kinase D at the Golgi contributes to ASC oligomerization, phosphorylation of NLRP3 at Ser-293, and release of NLRP3 from MAMs, resulting in the assembly of the mature NLRP3 inflammasome in the cytosol (17). These molecular episodes suggest that MAMs may be the location of NLRP3 inflammasome assembly.

Mitofusin 2 (MFN2), a mitochondrial outer membrane that regulates mitochondrial fusion within cells, facilitates the maintenance of cell homeostasis. MFN2 on the ER bridges ER and mitochondria by engaging in homotypic and heterotypic complexes with mitofusin 1 or 2 on the surface of mitochondria (18). Thus, MFN2 tethers ER to mitochondria implying that MFN2 participates in MAM establishment (18). Under RNA virus infection, MFN2 interacts with NLRP3, promoting NLRP3 recruitment to mitochondria and subsequently IL-1β secretion (9). However, it is unknown about the roles of mitochondria and MFN2 in MTB infection. Previous genome-wide association studies result and our study have shown highly expressed MFN2 in the TB group compared with the healthy control (HC) group (19). In this study, we first reported that mitochondrial MFN2 interacts and activates NLRP3 inflammasome and promotes IL-1β secretion upon MTB stimulation.

## Results

### mRNA microarray profiling of PBMCs is different between TBs and HCs

**Figure 1.**
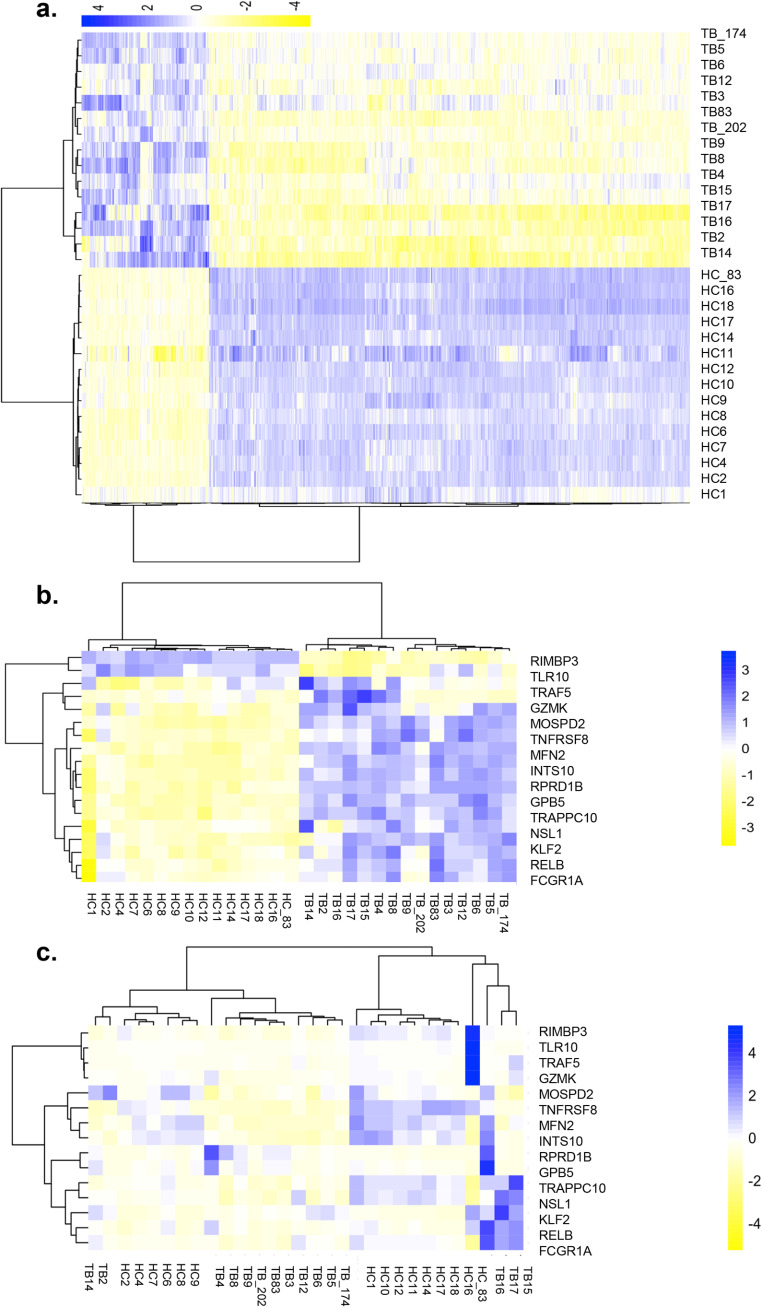
**Genome-wide mRNA profiling of PBMCs from TB patients (*TB*) and HC.** Cluster analysis of differentially expressed mRNAs. *a,* genome-wide mRNA profiles of PBMCs from TBs and HCs. The heatmap represents the results of a two-way hierarchical clustering of mRNA and samples. Each *row* represents an mRNA, and each *column* represents the sample tested. Cluster analysis of differentially expressed mRNAs is shown. The color key indicates the expression level of the mRNAs, *red* represents mRNAs with an expression level above the mean, and *green* represents mRNAs with an expression level below the mean. *b,* heatmap of different expression of 15 candidate genes (microarray). Each *row* represents an mRNA, and each *column* represents the sample tested. Cluster analysis of differentially expressed mRNAs is shown. The color key indicates the expression level of the mRNAs, *red* represents mRNAs with an expression level above the mean, and *green* represents mRNAs with an expression level below the mean. *c,* heatmap of different expression of 15 candidate genes (RT-qPCR). Each *row* represents an mRNA, and each *column* represents the sample tested. Cluster analysis of differentially expressed mRNAs is shown. The color key indicates the expression level of the mRNAs, *red* represents mRNAs with an expression level above the mean, and *green* represents mRNAs with an expression level below the mean.

To study the differential transcription profiling between TB patients and HCs, transcriptome microarray analysis was conducted to examine the gene expression profiling of peripheral blood mononuclear cell (PBMCs) from active TBs and HCs. All 18,853 mRNAs in the microarray were used to draw a heatmap of differential genes according to their expression levels. The genes that exhibited significant changes in expression were used for further validation. The TB group (*n* = 15) exhibited a significantly different profile compared with the HC (*n* = 15) group (Fig. 1*a*). A total of 1,595 differentially expressed mRNAs were identified between the two groups. Of these, 335 mRNAs were up-regulated (fold-change ≥ 2), and 1,260 mRNAs were down-regulated (fold-change ≥ 2).

We next determined GO annotation to infer the functions of the 1,595 differentially expressed mRNAs, which were classified into three categories: cellular component, molecular function, and biological process. The differentially identified mRNAs were subcategories into 35 hierarchically structured GO classifications (Table S1). The majority of mRNAs in the cellular component category had mitochondrial tricarboxylic acid cycle enzyme complex and mitochondrial related function. The biological process category showed regulation of natural killer cell and cytokine production involved in inflammatory function. This result indicated that the identified mRNAs involved in these GO categories might imply the most important roles in the TB infection process. To obtain more information of the differentially expressed mRNAs, pathway analysis was conducted using KEGG. Using a standard of *p* < 0.05 and impact factor threshold >0, the pathway analysis results demonstrated that several immune response pathways such as NOD−like receptor signaling pathway, cytokine−cytokine receptor interaction, and chemokine signaling pathway were enriched (Table S2), suggesting that the immune response was significantly associated with MTB infection.

The differentially expressed mRNAs between TBs and HCs were selected for rank function analysis to reveal the mRNAs with the prominent rank changes (△Rank) between two groups. By referring to our previous genome-wide association study result (19), and other published results (20), a total of 15 differentially expressed mRNAs were identified between the TB and HC groups, of which 8 were down-regulated and 7 were up-regulated (Table S3). These differentially expressed mRNAs were grouped as a biomarker panel, which distinguished TBs from HCs (Fig. 1*b*) and were confirmed by RT-qPCR (Fig. 1*c*). Heatmap images showed that hierarchical clustering of 15 identified mRNAs, where an increasing blue color shows increasing mRNA expression levels (Fig. 1*b*). Interestingly, from the differential mRNA expression profiling between TBs and HCs, a mitochondria outer member protein, MFN2, showed higher expression in TBs (Fig. 1, *b* and *c*). We calculated that MFN2 mRNA levels of 8 out of 15 TBs had 1.5-fold higher than the average MFN2 mRNA level of 15 HCs. The up-regulation of MFN2 during MTB infection has not been reported before, but is consistent with RNA virus infection (9), suggesting that MFN2 may take part in cell responses after pathogen infection.

### MTB stimulation up-regulates MFN2 expression and induces IL-1β secretion

**Figure 2.**
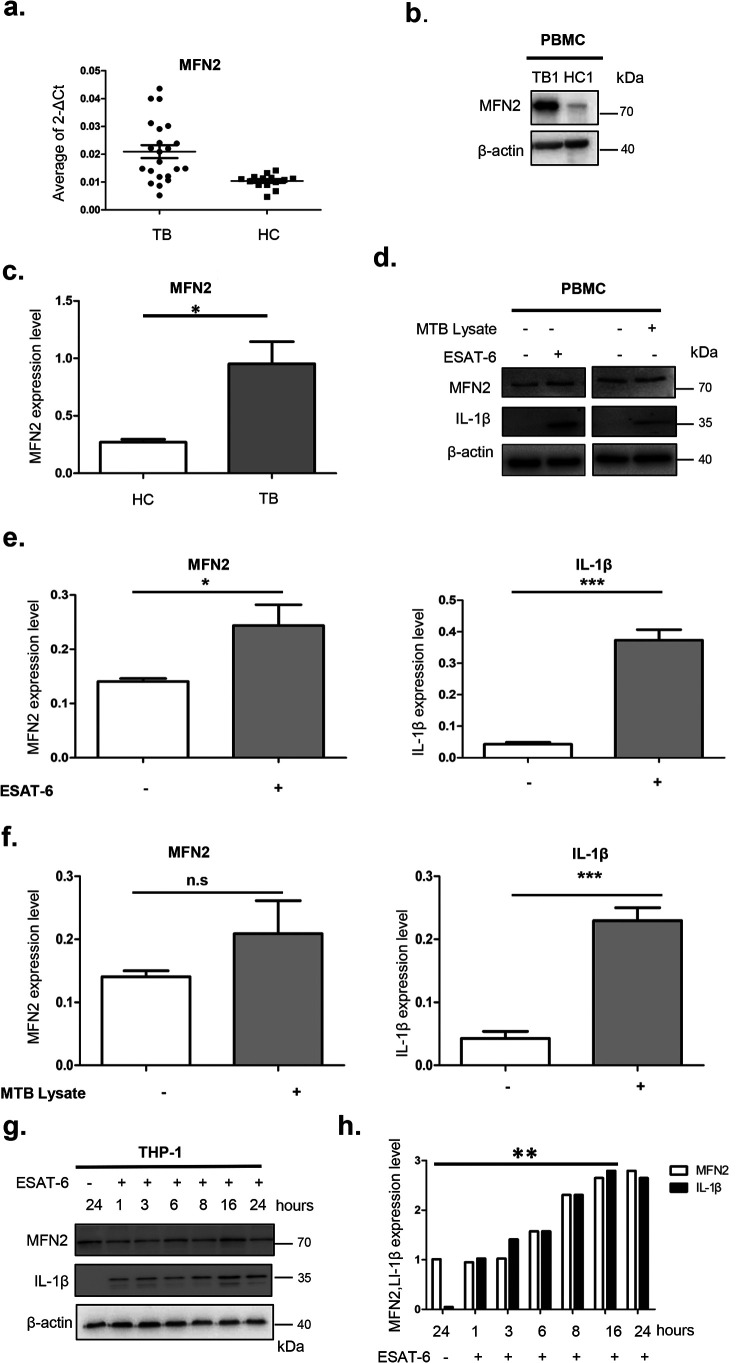
**MFN2 is up-regulated after MTB infection or stimulation.** *a,* RT-qPCR measurement of MFN2 mRNA isolated from PBMCs of active TB patients and HC. ** represents a significant difference between HC and TB, *p* < 0.01, as calculated by comparing the average fold-changes in gene expression of MFN2 in each TB patient (*n* = 20) sample with normalized average of the gene expression of each healthy control (*n* = 20). *b* and *c,* immunoblotting measurement of MFN2 expression of the PBMCs isolated from 5 TB patients and 3 healthy controls (image shows representative TB1 and HC1). β-Actin is a loading control. A *star sign* represents a significant difference between HC and TB, *p* < 0.05. *d–f*, immunoblotting measurement of MFN2 and IL-1β protein levels of the PBMCs isolated from 2 healthy controls with or without stimulation by heat-killed inactivated MTB lysates or ESAT-6 for 24 h. β-Actin is a loading control. *g* and *h,* immunoblotting measurement of MFN2 and IL-1β protein levels protein isolated from THP-1-marchophages stimulated by ESAT-6. MFN2 reached the highest level at 16 h after stimulation. Differences in the protein levels are compared with the previous time points. Images are representative of 3 independent experiments. Data are presented as mean ± S.D. (*n* = 3). *, *p* < 0.05; **, *p* < 0.01; ***, *p* < 0.001.

We further validated MFN2 mRNAs expression by real-time PCR and protein levels by immunoblotting in an independent sample set, which included additional 20 TB patients and 20 HCs. Total RNA and protein were extracted from PBMCs of active TBs and HCs. Our results showed that MFN2 mRNA expression was significantly up-regulated in TB patients (*p* < 0.05) (Fig. 2*a*), and the protein levels of MFN2 increased in TB patients (Fig. 2, *b* and *c*). Taken together, this implies that MFN2 may play a role in MTB infection.

To investigate whether MFN2 expression is induced after MTB challenge, we used two kinds of cells, PBMCs isolated from a healthy person and macrophages differentiated from the THP-1 cell line. These two kinds of cells were both stimulated by heat-inactivated MTB lysate and MTB antigen ESAT-6. We found that MFN2 expression was both up-regulated in PBMCs and THP-1 monocyte-derived macrophages upon MTB stimulation (including MTB lysate and ESAT-6) and IL-1β secretion was increased (Fig. 2, *d–f*). These results are consistent with that ESAT-6 promotes the secretion of IL-1β from THP-1 macrophages (10). In addition to this, we also found that MTB lysate reacted as an inducer of IL-1β secretion. Likewise, we found that after ESAT-6 stimulation of the THP-1 monocyte-derived macrophages, MFN2 protein levels and IL-1β secretion were increased in a time-dependent manner at the maximum 16 h post-stimulation (Fig. 2, *g* and *h*). These results suggest that MTB stimulation promotes the up-regulation of MFN2, further supporting our clinical observation of MFN2 increase in the TB group.

### MTB stimulation alters both mitochondrial membrane potential and MFN2 localization in THP-1 macrophages

**Figure 3.**
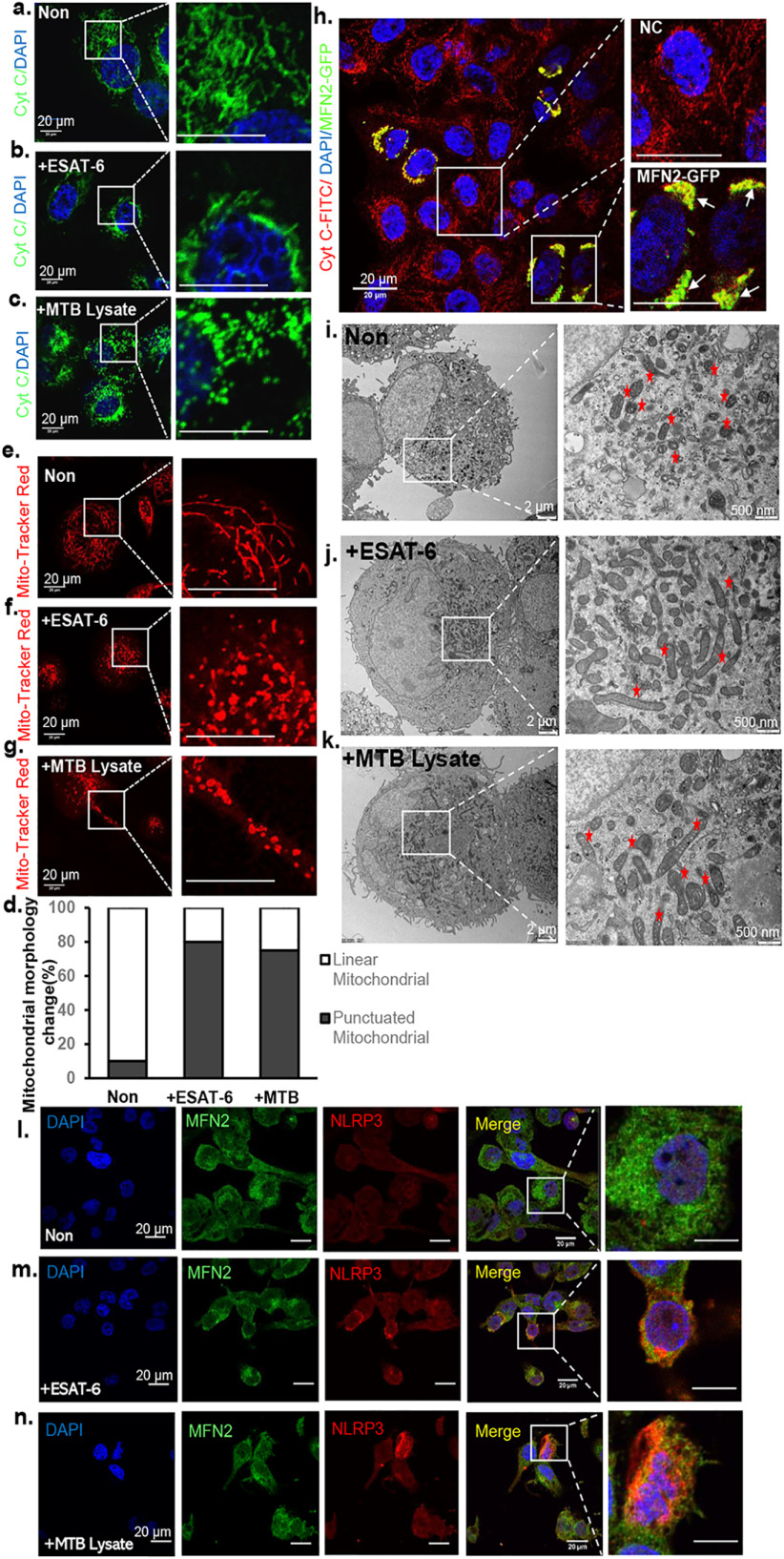
**MTB stimulation alters both mitochondrial membrane potential and MFN2 localization in THP-1 macrophages.** *a–c*, confocal immunofluorescent images of unstimulated THP-1 macrophages (*Non*), ESAT-6–stimulated macrophages (+*ESAT-6*), and MTB lysate-stimulated macrophages (+*MTB*). All these cells were fixed before staining with cytochrome *c* (*green*) antibody to label mitochondria, and DAPI to stain nuclei (*blue*). Note that cytochrome *c-*labeled mitochondria aggregate in perinuclear region. *Scale bar*, 20 μm. *d,* the percentage of the mitochondrial morphology change (from string mitochondria to punctuated mitochondria) with anti-cytochrome *c*. The number of punctuated mitochondria was counted from ESAT-6 or MTB-stimulated cells in at least 10 randomly chosen fields of view. 100 mitochondria from 10 macrophages in each group were used. The results are representative of 3 independent experiments. *e–g*, confocal immunofluorescent images of unstimulated THP-1 macrophages (*Non*), ESAT-6–stimulated macrophages (+*ESAT-6*), and MTB lysate–stimulated macrophages (+*MTB lysate*). Cells were stained with MitoTracker Red CMXRos (*red*) to label live-cell mitochondrial membrane potential. Note that most thread-like shapes (*e*) are changed to round shapes or donut shapes in *f* and *g*. *Scale bar*, 20 μm. *h,* images of THP-1macrophages transfected with MFN2-GFP. *Arrows* point to endogenous MFN2 colocalized with MFN2-GFP. *Scale bar*, 20 μm. *i–k,* representative TEM images of THP-1 macrophages stimulated with MTB lysate and ESAT-6 for 24 h. As a control, representative images of unstimulated cells are shown. The mitochondrial morphology in stimulated cells and their magnified images are shown (*white box* region and marked with a *red asterisk*). *Bar* indicates 2 (*left panel*) or 0.5 μm (*right panel*). *l–n,* confocal immunofluorescent images of unstimulated THP-1 macrophages, MTB lysate-stimulated macrophages, and ESAT-6–stimulated macrophages. All these cells were fixed before staining with MFN2 antibody (*green*) and NLRP3 antibody (*red*) to label endogenous MFN2 and NLRP3, respectively. DAPI (*blue*) was used to label nuclei. *Insets* show 6× magnified images of the *boxed region*. *Scale bar*, 20 μm.

To further investigate the involvement of mitochondria of macrophages during MTB stimulation, we conducted immunofluorescence analyses on THP-1 macrophages treated with MTB lysate or ESAT-6. Our results showed that after stimulation, the percentage of punctuated mitochondrial increased and a majority of mitochondria aggregated around the nuclei rather than dispersed in the cytosol (Fig. 3, *a–d*). We further investigated the changes in mitochondrial membrane potential after MTB stimulation in live cells. MitoTracker Red CMX Ros (*red*), which passively diffuses across the plasma membrane and accumulates in functional mitochondria, is an indicator of membrane potential. We observed that macrophage mitochondria were labeled by this dye and changed from string (thread) shape to dot or donut shapes after treatment with MTB lysate or ESAT-6, suggesting that the mitochondrial membrane potential was disrupted (Fig. 3, *d–f*). It has been reported that mitochondrial morphology and location changes are related to inflammatory responses. Here, we showed that MTB stimulation alters THP-1 macrophage mitochondria in both morphology and distribution. After stimulation, mitochondria changes from string shape to dot shape and aggregates (Fig. 3, *a–g*). Furthermore, TEM showed that the ultrastructure of mitochondria appeared in a hyperfusion pattern after MTB stimulation (Fig. 3, *i–k*). We speculated from these results that MTB stimulation causes mitochondria hyperfusion and leads to mitochondrial fragmentation, which is consistent with a previous report (21).

We then tested whether mitochondrial hyperfusion is caused by high expression of MFN2. THP-1 macrophages were transfected by a recombinant MFN2 plasmid to overexpress MFN2. We found that mitochondria had similar morphological changes from strings to dots with hyperfusion and aggregated around the nuclei (Fig. 3*h*). These data suggest that functional mitochondria and MFN2 may be involved in the inflammatory response upon MTB stimulation

The inflammasome is a protein complex involved in the activation of caspase-1 and IL-1β release. It has been reported that the MTB protein ESAT-6 is a potent activator of the NLRP3 inflammasome (10). We hypothesized that MFN2 may be involved in the NLRP3 inflammasome assembly and activation upon MTB stimulation. Using immunofluorescence analyses in MTB-stimulated macrophages, in contrast to unstimulated macrophages, the localization of NLRP3 aggregated around the nuclei, and colocalization of NLRP3 (*red*) and MFN2 (*green*) in mitochondria was observed after stimulation. Under resting conditions (unstimulated macrophages), the majority of NLRP3 was mainly located in the cytosol of THP-1 macrophages (Fig. 3, *l–n*). The perinuclear accumulation of mitochondria upon stimulation is suggestive of inflammasome assembly and activation. This observation agrees with other studies to show NLRP3 inflammasome localization on mitochondria and MAMs (14, 17). Taken together, our data suggest that MFN2 participates in the activation of NLRP3 inflammasome after MTB stimulation.

### MFN2 interacts with NLRP3 inflammasome

**Figure 4.**
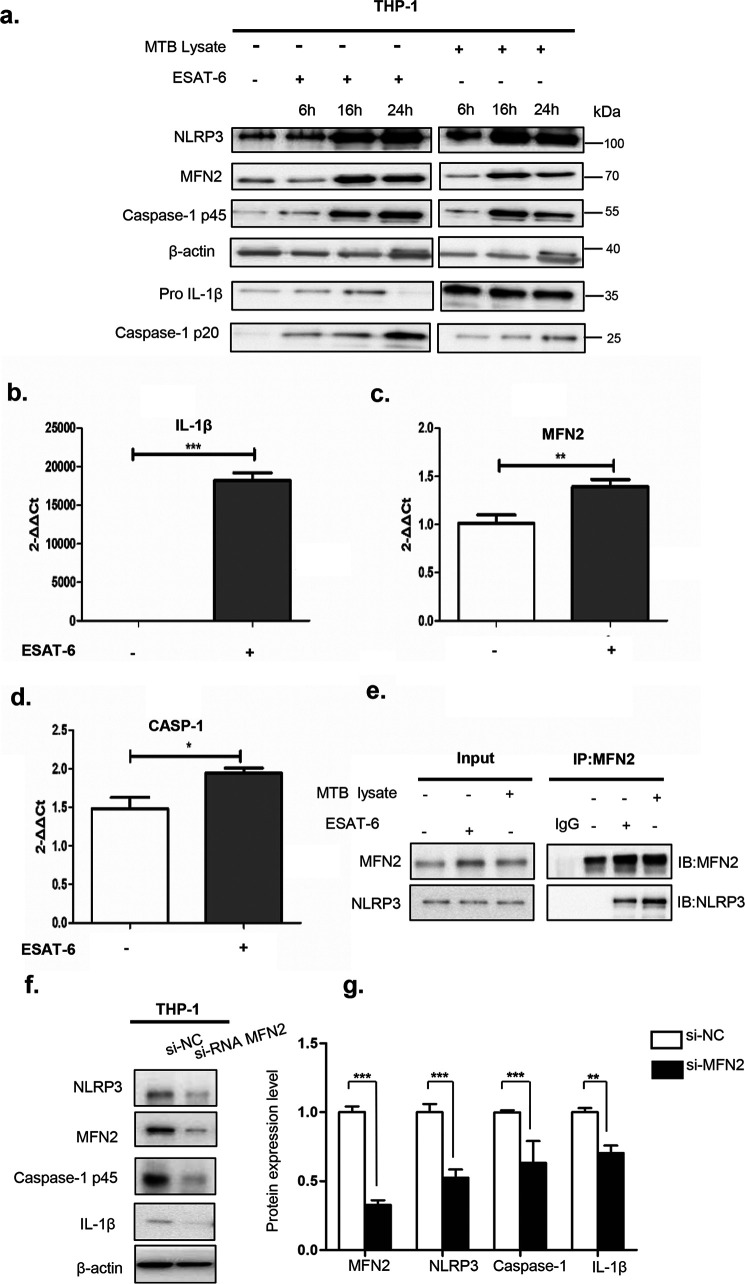
**Endogenous NLRP3 associates with MFN2 after stimulated with MTB.** *a,* immunoblotting analysis of lysates of THP-1 in cultured macrophages. Cell lysates prepared from macrophages following stimulation with MTB or ESAT-6 for 6, 16, and 24 h were analyzed by immunoblotting using anti-MFN2, anti-Caspase-1, anti-IL-1β, and anti-NLRP3 antibodies. β-Actin is a loading control. *b–d,* RT-qPCR analyses of THP-1 macrophages. ESAT-6–stimulated cells samples were collected at 16 h in parallel with immunoblotting analyses (*a*). *e,* endogenous immunoprecipitation (IP) from cell lysates with MFN2-specific antibody were analyzed by immunoblotting with mouse monoclonal antibodies against NLRP3 or MFN2. MTB- and ESAT-6–stimulated cells were collected in parallel with immunoblotting analysis. Stimulated cell samples were collected in the same way with immunoblotting analysis. Rabbit IgG is a negative control. *f* and *g*, samples from macrophages with transient expression of siRNA against MFN2 or EGFP control mRNAs (*si-NC*) were analyzed by immunoblotting after stimulated by MTB stimulation with antibodies against MFN2, Caspase-1, NLRP3, and IL-1β. β-Actin is a loading control. Images are representative of 3 independent experiments. Data are presented as mean ± S.D. (*n* = 3). *, *p* < 0.05; **, *p* < 0.01; ***, *p* < 0.001.

To further define whether MFN2 is involved in NLRP3 inflammasome activation, we conducted RT-qPCR and immunoblotting of cell lysates after MTB or ESAT-6 stimulation. As early as 6 h post-stimulation, NLRP3, MFN2, Caspase-1 p45, pro-IL-1β, and Caspase-1 p20 were up-regulated at protein expression levels, and at 16 h after stimulation their expression levels reached the highest except for that Caspase-1 p20 reached the highest protein expression level at 24 h (Fig. 4*a*). Also, we collected cell lysates after 16 h ESAT-6 stimulation and performed RT-qPCR to detect the inflammasome complex genes in the cell lysates of both stimulated and unstimulated groups. Two representative genes of the inflammasome complex (*IL-1B*, *CASP-1*) together with the *MFN2* gene were significantly increased after ESAT-6 stimulation (Fig. 4, *b–d*). These results suggest that ESAT-6 and MTB stimulation may activate the NLRP3 inflammasome and MFN2 likely plays a role in this process.

To confirm these observations, endogenous immunoprecipitation assays were conducted to monitor the interaction between MFN2 and NLRP3 in MTB-stimulated THP-1 macrophages. We collected cell lysates after 16 h MTB or ESAT-6 stimulation, endogenous NLRP3 was precipitated by antibodies to MFN2 in macrophages (Fig. 4*e*), suggesting the physical interaction between MFN2 and NLRP3. To further investigate whether MFN2 and NLRP3 function in a coordinated manner, we conducted siRNA MFN2 interference to knockdown MFN2 in THP-1 macrophages. Notably, MFN2 knockdown macrophages had a significantly reduced secretion of IL-1β and decreased active caspase-1 after being stimulated with MTB (Fig. 4, *f* and *g*). Collectively, these data suggest that MFN2 is required for the activation of NLRP3 inflammasome after MTB stimulation.

### MFN2 is involved in the assembly and activation of NLRP3 inflammasome during MTB infection

**Figure 5.**
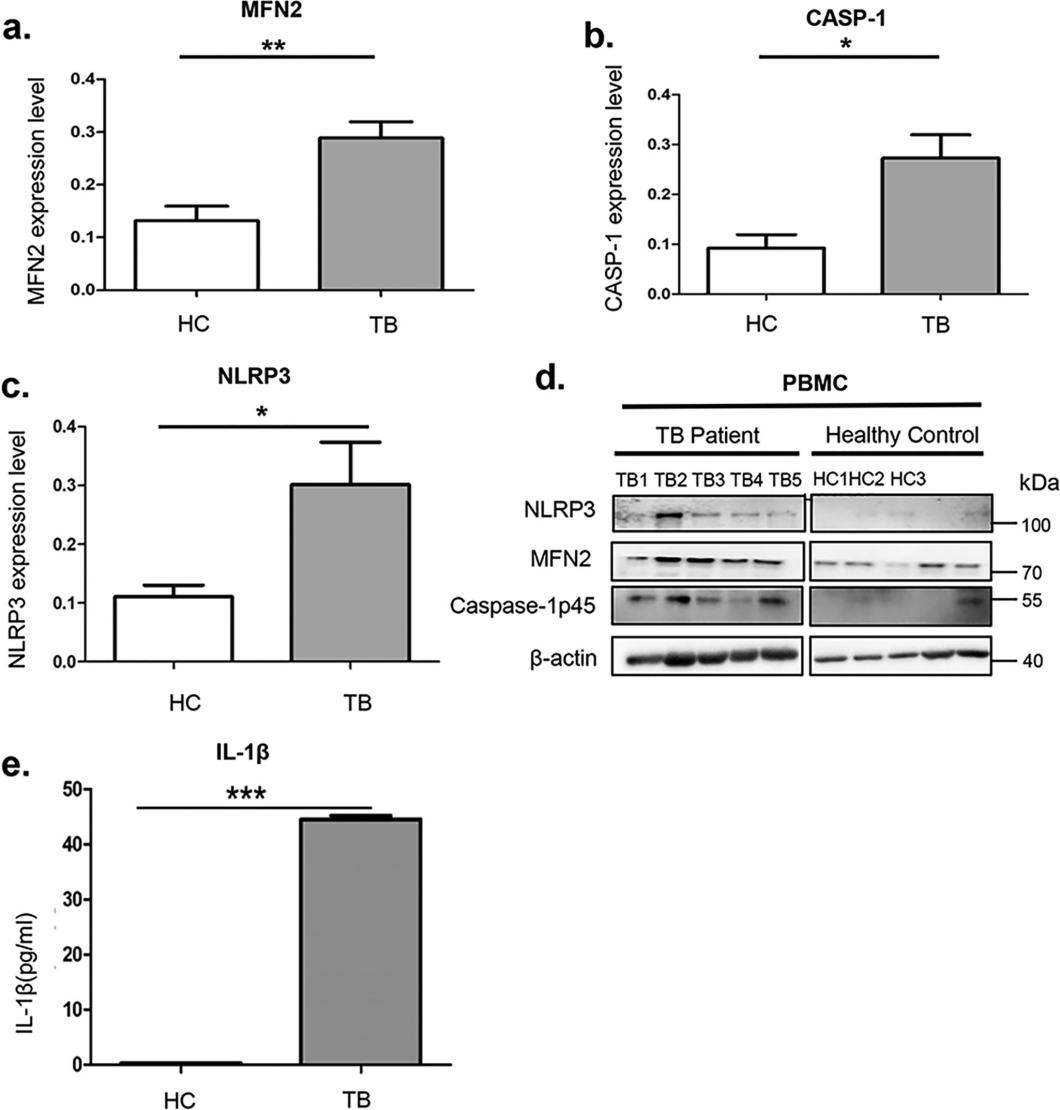
**Expression levels of MFN2 and NLRP3 inflammasome marker genes *in vivo*.** *a–d,* immunoblotting assays showed the endogenous levels of MFN2 (*a*), Caspase-1 p45 (*b*), and NLRP3 (*c*) isolated from PBMCs of active TB patients and PPD-skin test-negative healthy individuals with no prior exposure to TBs (HCs). β-Actin is a loading control. TB, *n* = 5; HC, *n* = 5. The experiments were repeated three times and representative blots are shown in *d*. *e,* ELISA measurements of IL-1β secretion of serum from the TBs and HCs. *, *p* < 0.05; **, *p* < 0.01; ***, *p* < 0.001.

To further confirm the involvement of NLRP3 inflammasome activation in human TB infection, PBMCs from 5 active TB patients and 5 healthy controls were isolated. The mRNA and protein levels of *NLRP3*, *CASP-1,* and *MFN2* were higher in the TB group (Fig. 5, *a–d*). Serum IL-1β levels in TBs were found significantly higher than HCs (Fig. 5*e*). These *in vivo* results confirm that MFN2 is required for the assembly and activation of the NLRP3 inflammasome after MTB infection.

## Discussion

Using transcriptome analysis, we found higher expression of MFN2 in a subset of 15 genes, which is capable of distinguishing TB patients from healthy controls. Different TB-specific subsets have been revealed such as a panel of three genes (*GBP5*, *DUSP3*, and *KLF2*) (22), and a combination of four genes (*GBP1, IFITM3, P2RY14*, and *ID3*) (23). A few of these differentially expressed genes (*e.g. GBP5* and *KLF2*) in TB patients are reproduced in our cohort, suggesting that genetic background of different populations and other unknown factors may have an impact on the candidate genes inferred from transcriptomic studies (24).

It has been shown that mitochondrion is involved in innate immunity. Pathogens modulate cellular pathways to adapt intracellular environment. The mitochondrial membrane protein MAVS plays an important role in the anti-virus pathway (25). Other mitochondrial membrane proteins may also be involved in regulating the host defense to pathogens (26). In this study, we found that MFN2, a well-known mitochondrial fusion protein, is a newly defined mitochondrial protein that is involved in the assembly and activation of NLRP3 inflammasome during MTB stimulation, resulting in IL-1β secretion for host immune defense. Up-regulation of MFN2 can impair mitochondria dynamics and morphology through recruiting NLRP3 to mitochondria outer member. These observations agree with other reports on the induction of pro-inflammatory cytokines such as IL-1β, IL-2, and tumor necrosis factor-α after MTB infection (27, 28). Thus, we have defined a new player in this TB pathogen-host interaction pathway upon MTB infection.

MFN2 regulates mitochondrial dynamics within cells. It has been reported that viruses, bacteria, and mycobacteria affect mitochondrial dynamics (9, 29, 30). Our results showed that after MTB stimulation, mitochondria changed from a classic string pattern to fragments or aggregates, which is typically present by enhanced fusion and the formation of long filamentous mitochondria is caused by up-regulation of MFN2. Other studies also show hyperfused mitochondria could affect viral replication by compromising MAMs integrity and signaling (31). Hyperfusion of mitochondria also leads to increased ROS generation, which may lead to activation of inflammasome (14). Moreover, it is proved that mitochondrial fusion is an indispensable step for activation of NLRP3 inflammasome (9). Fusion triggered perturbation in mitochondrial dynamics is associated with mitochondrial damage, clearance of damaged mitochondria via mitophagy. Mitophagy depends on the control of NLRP3 inflammasome activation (32). In summary, hyperfusion of mitochondria is important for host defense against pathogens and for inflammation response. However, whether mitophagy is involved in this process is still not clear, which is warranted for future investigation.

Mitochondria play important roles in MTB and other bacterial infection, in which MTB and other pathogenic bacteria-secreted proteins trigger structural changes in mitochondria to modulate their functions (33). Virulent strains of MTB inhibit apoptosis in macrophages through up-regulation of anti-apoptotic proteins, disruption of mitochondrial transmembrane potential, and depletion of cytochrome *c* (34). H37Rv, a pathogenic strain of MTB, promotes necrosis by inducing substantial alterations to mitochondrial transmembrane potential to evade host defenses and exit macrophages to disseminate to and infect the surrounding cells (35). A recent report revealed that virulent MTB infection affects mitochondria distribution, size, number, and fragmentation, whereas nonpathogenic strains of mycobacteria were unable to elicit the same effects (36), suggesting specific virulence factors are required for this process. Similarly, our results showed that MTB and virulence factors ESAT-6 induced changes of mitochondrial morphology and MFN2 localization, which may contribute to TB infection.

**Figure 6.**
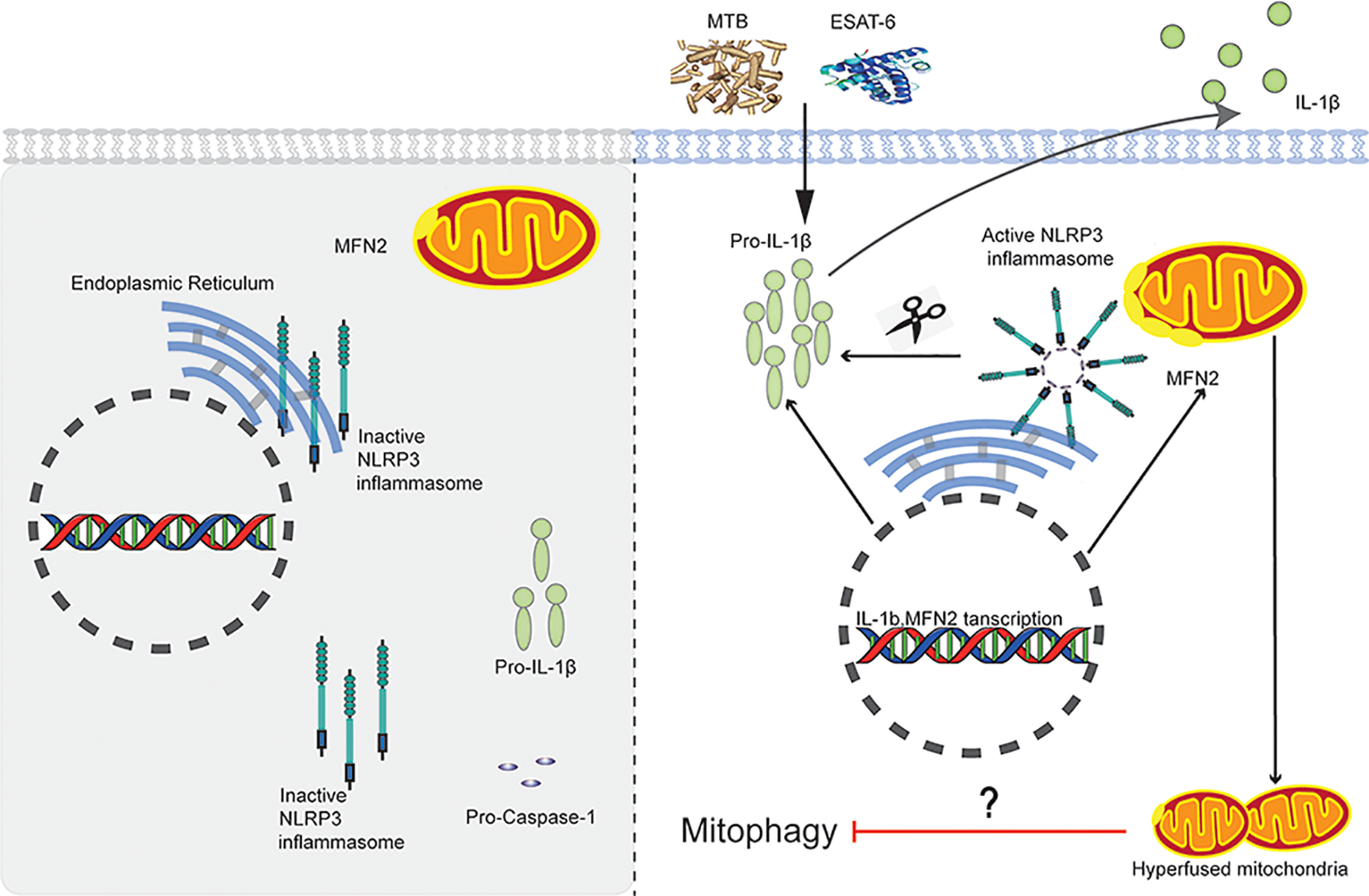
**A Proposed model of MFN2-dependent activation of the NLRP3 inflammasome during MTB infection.** After stimulated with MTB or ESAT-6, MFN2 promotes the association of NLRP3 and MFN2 through procapase-1 to form the NLRP3 inflammasome at mitochondrial outer member associated with endoplasmic reticulum membranes (MAMs). The NLRP3 inflammasome cleaves the pro-IL-1β to produce its mature form IL-1β for secretion.

The inflammasomes are multiprotein complexes sensing tissue damage and infectious agents to initiate innate immune responses. It is well-known that MTB triggers IL-1β production in infected macrophages as a consequence of NLRP3-inflammasome activation *in vitro* (10, 37, 38). The underlying mechanism of which NLRP3-inflammasome is activated during MTB infection is poorly understood. A previous study has demonstrated a MFN2-dependent NLRP3-inflammasome activation and IL-1β production after RNA virus infection (9). We here demonstrated that mitochondrial change upon MTB stimulation was an important episode during NLRP3 activation. Mitochondria physically contact and interact with ER at MAMs, which suggests the location of NLRP3 inflammasome assembly (16). Our biochemical assays further demonstrated that the majority of NLRP3 was localized to the ER and cytosol of THP-1 cells. Upon MTB stimulation, NLRP3 and MFN2 colocalized and aggregated in perinuclear region, suggesting that NLRP3 was recruited by MFN2 and translocated on MAMs for inflammasome assembly (a proposed model in Fig. 6). This expands the knowledge on the molecular and cellular mechanism of MFN2-dependent NLRP3 inflammasome assembly.

In summary, we have defined that MFN2 is involved in the assembly and activation of NLRP3 inflammasome during TB infection. These findings highlight the important roles of MFN2 and mitochondria in the NLRP3-IL-1β inflammatory pathway.

## Experimental procedures

### Subjects

All the subjects recruited in this study were Han Chinese. The diagnosis of TB was according to the previously published criteria (19): (i) etiology or pathology results (acid-fast bacilli staining or culture); (ii) clinical presentation (symptoms or signs); (iii) imaging findings (chest radiography or computed tomography scan); (iv) contact history (family and close contact); (v) purified protein derivative (PPD) skin tests or interferon γ release assay positive results; (vi) positive clinical response to anti-TB therapy; and (vii) exclusion of other diseases, such as pneumonia, tumor, and inflammatory diseases. Clinical TB could be diagnosed when features of (ii) clinical presentation and (iii) imaging findings plus either two of (iv) contact history to (vii) exclusion of other diseases were present. Confirmed TB diagnosis is made when positive of (i) etiology or pathology results plus (ii) clinical presentation and/or (iii) imaging findings.

Healthy controls were children in comparable age and sex to TB patients with negative tuberculin skin tests (TST) and T-SPOT.TB tests for TB-specific T lymphocytes, normal chest radiograph and no clinical symptoms of diseases. Individuals with positive HIV, positive hepatitis B virus (HBV) or hepatitis C virus (HCV), diabetes, malignancies, severe autoimmune diseases, and those who took immunosuppressive or immunopotentiator pills, or were in pregnancy or lactation were excluded.

In the microarray study, 30 subjects were enrolled within two groups: active TB (n = 15) and HCs (*n* = 15). An additional 20 active TB patients, and 20 HCs were enrolled for further qPCR validation. The demographic characteristics of all participants in this study are shown in Table S4. This study was performed in accordance with the guidelines of the Helsinki Declaration and was approved by the Ethics Committee of Beijing Children's Hospital, Capital Medical University (number 2015-31).

### Isolation of peripheral blood mononuclear cells

Peripheral blood samples (2 ml) were collected in heparin-containing vacutainer tubes from each subject. PBMCs were separated by density gradient using Lymphocyte Cell Separation Media (Tianjin Haoyang Biological Manufacture Co., China) within 6 h of blood collection. The number of live cells were counted using Muse Cell Analyzer (Merck & Millipore, Germany).

### RNA extraction

Total RNA was extracted from PBMCs and THP-1 cells (ATCC, USA) by using the miRneasy^®^ Mini kit (Qiagen, Germany) according to the protocols recommended by the manufacturer. RNase-free DNase I (Qiagen) was used to remove the genomic DNA contamination. The integrity and quality of RNA was evaluated using an Agilent 2100 Bioanalyzer (Agilent Technologies, USA). RNA with a 2100 RNA integrity number ≥7.0 and 28S/18S > 1.0 was used for the microarray study and quantitative PCR validation.

### Microarray assays and bioinformatics analysis

RNA samples from each group were used to generate fluorescence labeled cRNA targets for the Agilent Whole Human Genome Oligo Microarray (4 × 44 K, including ∼41,000 genes and transcripts). Labeled cRNA targets were then hybridized with the slides. After hybridization, slides were scanned on the Agilent Microarray Scanner (Agilent Technologies). Data were extracted with Feature Extraction software 10.7 (Agilent Technologies). Raw data were normalized by the Quantile algorithm, Gene Spring Software 12.6.1 (Agilent Technologies). The microarray experiments were performed by following the protocol of Agilent Technologies at Shanghai Biotechnology Corporation. The microarray data were deposited in GEO database accession number GSE98461. Fold-changes of gene expression values were calculated in 2 pairwise comparisons. Differentially expressed genes were identified and selected for further analysis based on the *p* value < 0.05 and with a fold-change of at least 2 or more. The selected genes were grouped in functional categories based on Gene Ontology database (RRID:SCR_002811), and functional pathways (KEGG) were also analyzed by using the online SAS analysis system.

### Quantitative real-time PCR analysis

To verify microarray data from PBMCs and analyze the expression levels of *CASP-1, IL-1B, MFN2*, and *NLRP3* genes from THP-1 macrophages, total cellular RNA, and subsequent complementary DNAs were prepared. A total of 200 ng of purified RNA was reverse transcribed to cDNA using iScript cDNA Synthesis (Bio-Rad, USA) according to the manufacturer's protocol. SYBR Green (Power SYBR Green PCR Master Mix, Applied Biosystems Inc., USA) binding in dsDNA was measured using the ABI 7900 Real-time PCR System (Applied Biosystems Inc.). The following primer sets were used for RT-PCR: *CASP-1* forward, 5′-GTCACTGAGGTCCATCTGAAC; reverse, 5′-CATCCACTCCTGGAAGAACCT; *IL-1B* forward, 5′-CAGCCAATCTTCATTGCTCA; reverse, TCGGAGATTCGTAGCTGGAT; *MFN2* forward, 5′-CTCTCGATGCAACTCTATCGTC; reverse, 5′-TCCTGTACGTGTCTTCAAGGAA; *NLRP3* forward, 5'-TCACAACTCGCCCAAGGAGGAA; reverse, 5′-AGAGACCACGGCAGAAGCTAG. We calculated 2-ΔΔ*C_T_* and used this statistic to determine relative gene expression. The reference gene was *GAPDH*.

### Cell culture, stimulation, and transfection

PBMCs were cultured with AIM-V (Invitrogen Life Technologies, USA) containing 2 mm l-glutamine, 50 μg/ml of streptomycin sulfate, 10 μg/ml of gentamicin sulfate. THP-1 cells were cultured overnight with RPMI 1640 (Gibco, USA) containing 10% fetal bovine serum, 100 ng/ml of tetradecanoyl phorbol acetate. The following day, the original medium was replaced with fresh tetradecanoyl phorbol acetate-free medium and maintained for an additional 48 h to ensure that the cells reverted to a resting macrophage phenotype before infection. All cells were stimulated with 5 μg/ml of purified MTB-specific antigen ESAT-6 (Abcam, USA) or 50 ng/ml of lysate of MTB standard strain H37Rv (gift from Prof. Zongde Zhang) at 37 °C in a humidified incubator with 5% CO_2_. THP-1 macrophages were transfected with pMFN2-GFP plasmids constructed in our laboratory by using Lipofectamine 3000 (Thermo Fisher, USA) according to the manufacturer's instructions.

### siRNA transfection in THP-1 macrophages

THP-1 macrophages (5 × 10^5^ cells in 6-well–plates) were transiently transfected with siRNA targeting *MFN2* (Syngen Tech, Beijing, China) or negative control siRNA (Syngen Tech, Beijing, China) using RNA iMAX transfection reagent (ThermoFisher) following the manufacturer's instructions. All experiments were performed 24 h after transfection. The specific siRNAs targeting *MFN2* were designed and synthesized by Syngen Tech (Syngen Tech, Beijing, China), and the most effective single siRNA was used for further experiments as following: siMFN2, GGACCUCCAUGGGCAUUCUUGUUGU; siNC, UUCUCCGAACGUGUCACGU.

### Immunoblotting

Whole cell lysates were separated by SDS-PAGE and transferred onto Immobilon-P transfer membrane (Merck Millipore). The immunoblotting was probed with the following specific primary antibodies overnight at 4 °C: anti-Mitofusin-2 (1:500, CST, USA), anti-NLRP3 (1:500, CST), anti-caspase-1 (1:1000, CST), anti-IL-1β (1:500, CST), and anti-β-actin (1:20,000, Sigma, USA). After washing with PBS-T, the membrane was incubated with each corresponding secondary antibody for 1 h at room temperature. Visualization was performed using an ECL Plus detection system (Thermo Scientific, USA) and Sage Brightness ECL (Sage Creation, Beijing, China). The intensity of protein expression was quantified by ImageJ software.

### Mitochondrial membrane potential measurement

THP-1 macrophages were incubated with 5 μm MitoSOX™ Red (Invitrogen) for 15 min at 37 °C and protected from the light, washed with PBS. Confocal images were acquired using a ×100 oil objective with NA 1.40 on a Nikon confocal microscope (ECLIPSE Ti-C2, Japan). Images were obtained using the NIS-Elements AR 3.2 software provided by Nikon (Japan).

### Immunofluorescence microscopy

THP-1 macrophages were grown in 35-mm plates with ESAT-6 or MTB lysate stimulation for 16 h. Preconditioned cells were washed three times with PBS slowly for 3 min each and then fixed with 4% paraformaldehyde for 15 min, and washed three times with PBS (10 min each). The cells were treated with 0.5% Triton X-100 for 15 min, washed three times with PBS (5 min each), and then treated with 5% BSA for 1 h. Cells were incubated with anti-Mitofusin-2 (1:100), anti-NLRP3 (1:300), anti-cytochrome *c* (1:1000) antibodies overnight at 4 °C, The nuclei were stained with 4',6'-diamidino-2-phenylindole (DAPI). After three rinses, fluorescein isothiocyanate-conjugated secondary antibodies were used to visualize the proteins by fluorescence microscopy (Nikon confocal microscope, ECLIPSE Ti-C2, Japan). Images were obtained using the NIS-Elements AR 3.2 software provided by Nikon.

### Transmission EM (TEM)

For TEM analysis, cells were fixed with 2.5% (v/v) glutaraldehyde with phosphate buffer (0.1 m, pH 7.4), washed four times in phosphate buffer at 4 °C. Cells were postfixed with 1% (w/v) osmium tetroxide (OsO_4_) and 1.5% (w/v) potassium ferricyanide aqueous solution at 4 °C for 2 h, dehydrated through a graded ethanol series (30, 50, 70, 80, 90, 100, and 100%, 5 min each at 4 °C) into pure acetone (2 × 5 min). Samples were infiltrated in a graded mixture (3:1, 1:1, 1:3) of acetone and SPI-PON812 resin (16.2 ml of SPI-PON812, 10 ml of dodecenyl succinic anhydride and 8.9 ml of methyl nadic anhydride, then changed to pure resin. Finally, cells were embedded in pure resin with 1.5% N,N-Dimethylbenzylamine and polymerized for 12 h at 45 °C, 48 h at 60 °C. The ultrathin sections (70 nm thick) were sectioned with microtome (Leica EM UC7), double-stained by uranyl acetate and lead citrate, and examined by a transmission electron microscope (FEI Tecnai Spirit120kV).

### Endogenous immunoprecipitation (IP)

Protein A/G-agarose beads were washed for 2 times with PBS and resuspended with ice-cold 100 mm NETN (20 mm Tris, 100 mm NaCl, 1 mm EDTA·2Na, 0.5% Octylphenoxypolyethoxyethanol (NP-40)). An appropriate amount of primary antibody was added to 500 μl total volume. The mixture was placed on a low-speed rotating shaker for 2 h at 4 °C and the beads were collected by centrifugation (1000 × *g*, 5 min). Harvest was with ∼1 × 10^6^ cells by centrifugation at 1000 rpm for 3 min. The cell pellet was resuspended with 1 ml of ice-cold PBS and transferred to a 1-ml centrifuge tube for centrifugation at 1000 rpm for 3 min at 4 °C. PBS was replaced by ice-cold NETN buffer. The resuspended pellet was placed on a low-speed rotating shaker for 20 min at 4 °C, then centrifuged at the highest speed (13,000 rpm) for 10 min at 4 °C. The supernatant was transferred to new tubes. The antigen-antibody complex solution was slowly shanked on a rotating shaker at 4˚C for overnight. Immunoblotting was performed as described before.

### Antibodies

Primary antibodies used in this study include the following: rabbit polyclonal anti-caspase-1 (CST), rabbit polyclonal anti-IL-1β (CST), rabbit polyclonal anti-Mitofusin-2 (CST), mouse anti-Mitofusin-2 antibody (Abcam), rabbit polyclonal anti-NLRP3 antibody (CST), mouse anti-β-actin (Sigma-Aldrich), rabbit monoclonal anti-TOM20 (BD), rabbit polyclonal anti-cytochrome *c* (Cell Signaling), and normal rabbit IgG (Cell Signaling).

The secondary antibodies used for Western blotting analysis were HRP-conjugated anti-mouse IgG (ZhongShanJinQiao), HRP-conjugated anti-rabbit IgG (ZhongShanJinQiao), and HRP-conjugated anti-goat IgG (ZhongShanJinQiao).

### Statistical analysis

Each experiment was independently repeated at least three times. The data were analyzed using Student's *t* test. *p* < 0.05 was regarded as a statistically significant difference.

## Data availability

All data are contained within the manuscript. The ChIP-seq and RNA-seq data have been deposited in Gene Expression Omnibus under accession number GSE98461.

## Supplementary Material

Supporting Information
