# Supplementary material for: Mycobacterium tuberculosis infection up-regulates MFN2 expression to promote NLRP3 inflammasome formation
Source: J Biol Chem. 2020 Oct 16;295(51):17684–97. doi: 10.1074/jbc.RA120.014077 (PMC7762945; doi:10.1074/jbc.RA120.014077)
Supplement: Supporting Information [file supp_295_51_17684__index.html]

Mycobacterium tuberculosis infection upregulates MFN2 expression to promote NLRP3 inflammasome formation — MFN2 induces NLRP3 inflammasome — Mycobacterium tuberculosis infection up-regulates MFN2 expression to promote NLRP3 inflammasome formation — MFN2 induces NLRP3 inflammasome — Supporting Information 

# *Mycobacterium tuberculosis* infection up-regulates MFN2 expression to promote NLRP3 inflammasome formation

## Supporting Information

- Supplementatary Tables 1-4 - Table S1 to Table S4
