# Supplementary material for: Mycobacterium tuberculosis infection up-regulates MFN2 expression to promote NLRP3 inflammasome formation
Source: J Biol Chem. 2020 Oct 16;295(51):17684–97. doi: 10.1074/jbc.RA120.014077 (PMC7762945; doi:10.1074/jbc.RA120.014077)
Supplement: Supporting Information [file supp_RA120.014077_160435_2_supp_609043_qhvyyv.pdf]

Supplementary Tables

Table S1. Gene Ontology (GO) classification of differentially expressed mRNAs by transcriptome analysis between tuberculosis patients and healthy controls.

ID	Description	*p*-value	Gene ID	TYPE	Enrich factor
GO:0010340	carboxyl-O-methyltransferase activity	0.0002442	*ICMT/PCMT1/PCMTD2/ARMT1*	Molecular function	10.2071
GO:0051998	protein carboxyl O-methyltransferase activity	0.0002442	*ICMT/PCMT1/PCMTD2/ARMT1*	Molecular function	10.2071
GO:0006824	cobalt ion transport	0.0024033	*SLC11A2/SLC30A5/TCN2*	Biological process	9.33172
GO:0010990	regulation of SMAD protein complex assembly	0.0024033	*PPM1A/PMEPA1/LDLRAD4*	Biological process	9.33172
GO:0010991	negative regulation of SMAD protein complex assembly	0.0024033	*PPM1A/PMEPA1/LDLRAD4*	Biological process	9.33172
GO:0072223	metanephric glomerular mesangium development	0.0024033	*EGR1/PDGFB/PDGFRB*	Biological process	9.33172
GO:0000828	inositol hexakisphosphate kinase activity	0.0025152	*PPIP5K2/IP6K2/IP6K1*	Molecular function	9.18639
GO:0000832	inositol hexakisphosphate 5-kinase activity	0.0025152	*PPIP5K2/IP6K2/IP6K1*	Molecular function	9.18639
GO:0052723	inositol hexakisphosphate 1-kinase activity	0.0025152	*PPIP5K2/IP6K2/IP6K1*	Molecular function	9.18639
GO:0052724	inositol hexakisphosphate 3-kinase activity	0.0025152	*PPIP5K2/IP6K2/IP6K1*	Molecular function	9.18639
GO:0005947	mitochondrial alpha-ketoglutarate dehydrogenase complex	0.0029036	*BCKDK/ DBT/BCKDHB*	Cellular component	8.74212

GO:0032364	oxygen homeostasis	0.0045768	*ALAS2/HIF1A/EGLN1*	Biological process	7.77644
GO:0019763	immunoglobulin receptor activity	0.0047862	*FCER1A/MS4A2/FCGR1B*	Molecular function	7.65533
GO:0046974	histone methyltransferase activity (H3-K9 specific)	0.0047862	*PRDM8/SUV39H1/SETDB2*	Molecular function	7.65533
GO:0055131	C3HC4-type RING finger domain binding	0.0047862	*HSPA1A/HSPA8/KCNH2*	Molecular function	7.65533
GO:0030062	mitochondrial tricarboxylic acid cycle enzyme complex	0.0055111	*BCKDK/DBT/BCKDHB*	Cellular component	7.2851

Table S2. KEGG enrichment analysis of the differential expressed mRNAs between tuberculosis patients and healthy controls.

ID	Description	*p*-value	Gene ID	Count	Enrich factor
hsa00730	Thiamine metabolism	0.028482	*THTPA/NTPCR*	2	6.901639
hsa03060	Protein export	0.004876	*SEC63/IMMP1L/SRPRB/SRP68/SEC62/SPCS2*	6	3.600855
hsa00071	Fatty acid degradation	0.010851	*ECI2/ADH5/ACSL1/ACSL6/HADH/ACADM/ ACADSB/ACOX1*	8	2.568052
hsa04612	Antigen processing and presentation	0.001237	*CTSL/HSPA1A/HSPA1L/HSPA8/KIR2DS4/KIR3DL1/KIR3DL2/ KLRC1/KLRD1/NFYA/NFYB/KIR2DL5A/ RFXAP/TNF*	14	2.477512
hsa05020	Prion diseases	0.042512	*EGR1/HSPA1A/IL1B/ NCAM1/PRKACB/ C1QB*	6	2.300546
hsa04146	Peroxisome	0.004055	*ECI2/PXMP4/ACSL1/ABCD2/ACSL6/MLYCD/MVK/ACOX1/FAR2/ PXMP2/NUDT12/PEX3/PEX11A*	13	2.271426
hsa05332	Graft-versus-host disease	0.033114	*IL1B/KIR3DL1/ KIR3DL2/KLRC1/ KLRD1/KIR2DL5A/TNF*	7	2.247045
hsa03022	Basal transcription factors	0.04777	*GTF2A1/GTF2E1/ GTF2F1/GTF2H1/ GTF2H3/TAF4*	6	2.23837
hsa00280	Valine, leucine and isoleucine degradation	0.037024	*DBT/HADH/ACADM/ ACADSB/ALDH6A1/ AUH/BCKDHB*	7	2.195976
hsa03018	RNA degradation	0.012393	*C1D/TOB2/BTG3/DCP1B/CNOT1/SKIV2L2/CNOT2/DCP1A/EXOS C5/EDC3/WDR61*	11	2.138536

hsa04621	NOD-like receptor signaling pathway	0.024841	*CARD8/PYDC1/CXCL2/IL1B/CXCL8/NLRC4/NOD2/MAP3K7/TNF*	9	2.105585
hsa04060	Cytokine-cytokine receptor interaction	6.81E-05	*CCR9/IL24/CCR5/CX3CR1/IFNLR1/CLCF1/IL17RA/CXCR3/CXCL2/CXCL3/IFNAR1/IL1B/IL2RB/IL7/CXCL8/CXCR2/IL15/KIT/CCL4L1/TNFRSF12A/PDGFB/PDGFRB/CCL28/ACKR3/TNFRSF17/CCL3/CCL3L1/CCL4/CCL20/BMPR1A/XCL2/TNF/CCR2/RELT/CD27/CCL4L2/CD40LG*	37	1.92725
hsa04650	Natural killer cell mediated cytotoxicity	0.035968	*SH2D1B/KLRK1/NCR3/IFNAR1/KIR2DS4/KIR3DL1/KIR3DL2/KLRC1/KLRD1/NFATC2/NFATC3/KIR2DL5A/ SOS1/TNF/PIK3R3/NCR1*	16	1.623915
hsa04062	Chemokine signaling pathway	0.017508	*CCR9/ADCY9/CCR5/CX3CR1/CXCR3/CXCL2/CXCL3/CXCL8/CXCR2/CCL4L1/PRKACB/CCL28/RAP1A/CCL3/CCL3L1/CCL4/CCL20/SOS1/ XCL2/CCR2/PIK3R3/ CCL4L2*	22	1.606731
hsa04010	MAPK signaling pathway	0.030225	*GADD45G/DUSP10/DDIT3/DUSP7/DUSP8/FGF3/FGF7/HSPA1A/HSPA1L/HSPA8/IL1B/JUND/MAPT/MEF2C/NFATC2/PDGFB/PDGFRB/PPM1A/PRKACB/CACNA2D3/TAOK1/RAP1A/CACNG6/RELB/SOS1/MAP3K7/TNF/MAP4K3*	28	1.442134

Table S3. 15 different expression mRNAs between tuberculosis patients (TB, n=15) and healthy controls (HC, n=15) in microarray set.

**mRNA (Gene Symbol)**	***p*-value**	**Fold Change (TB vs. HC)**	**Expression Level**
RIMBP3	7.7E-14	0.240351	down
TLR10	5.77E-09	0.256645	down
TRAF5	2.35E-08	0.346212	down
GZMK	0.000139	0.414256	down
MOSPD2	6.06E-05	0.416715	down

TNFRSF8	0.007942	0.621902	down
NSL1	0.000629	0.762981	down
KLF2	0.007107	0.830859	down
MFN2	0.042893	1.377347	up
INTS10	3.54E-15	2.019486	up
RPRD1B	4.64E-06	2.221996	up
TRAPPC10	3.7E-11	2.561061	up
GBP5	0.027499	2.689441	up
RELB	2.74E-10	3.079927	up
FCGR1A	0.03695	3.558353	up

Table S4. Demographic characteristics of the participants in this study.

**Study complex**	**Characters**	**TB**	**HC**	***p*-value**
Microarray set	n	15	15	
	Male/Female	10/5	9/6	0.43
	Age (median, range)	6.5 (2-10)	6.15(3-10)	0.28

TB: tuberculosis, HC: healthy control.
